# Editorial: Long-acting growth hormone therapy in pediatric growth hormone deficiency

**DOI:** 10.3389/fendo.2026.2005952

**Published:** 2026-10-08

**Authors:** Anastasia Ibba, Giuseppa Patti, Sandro Loche

**Affiliations:** 1 Pediatric Endocrinology Unit and Newborn Screening Centre, Pediatric Hospital Microcitemico “A. Cao”, Azienda Ospedaliera di Rilievo Nazionale e di Alta Specializzazione (ARNAS) Brotzu, Cagliari, Italy; 2 Department of Pediatrics, Istituto di Ricovero e Cura a Carattere Scientifico (IRCCS) Istituto Giannina Gaslini, Genova, Italy; 3 Department of Neuroscience, Rehabilitation, Ophthalmology, Genetics, Maternal and Child Health - University of Genova, Genova, Italy; 4 Research Area for Innovative Therapies in Endocrinopathies, Bambino Gesù Children Hospital, Istituto di Ricovero e Cura a Carattere Scientifico (IRCCS), Rome, Italy

**Keywords:** growth hormone, long acting GH, Noonan syndrome, pediatric growth hormone deficiency, short stature

Growth hormone deficiency (GHD) in children and adolescents is a condition that may require long-term therapy with recombinant human growth hormone (rhGH). The efficacy and safety of rhGH have been documented in several large, long-term observational registries. However, treatment outcomes vary considerably among patients, as several factors may influence the response to therapy. Greater severity of GHD, younger age, prepubertal status and treatment-naïve status at treatment initiation, better adherence, and longer treatment duration are all associated with better outcomes.

To improve adherence, weekly long-acting growth hormone (LAGH) formulations have been developed and approved by the European Medicines Agency (EMA) and the US Food and Drug Administration (FDA) for the treatment of childhood GHD. Short-term studies have demonstrated efficacy and safety comparable to those of daily rhGH therapy (1). However, real-world data remain limited. Daily injections have been the standard of care for more than three decades, yet treatment adherence has consistently represented one of the major challenges to achieving optimal outcomes. LAGH formulations seek to address this limitation by reducing injection frequency while maintaining efficacy and safety.

The articles presented in this Research Topic collectively illustrate the real-world implementation of LAGH, expert consensus on clinical practice, insights into the differential diagnosis of short stature, and reflections on the broader implications of reducing treatment burden. Together, these contributions highlight the progressive integration of LAGH into routine clinical care and its potential application not only in GHD but also in other growth disorders, while emphasizing the need to balance therapeutic innovation with robust clinical evidence.

Tamaro et al. presented a retrospective study evaluating the real-world experience with somatrogon in children with GHD. Clinical trials have already demonstrated the non-inferiority of weekly somatrogon compared with daily GH in pediatric GHD, but observational studies are critical for understanding its performance under routine clinical conditions. Their experience suggests that the effectiveness observed in controlled trials can largely be replicated in clinical practice while maintaining an acceptable safety profile. Equally important, real-world evidence captures practical aspects—including treatment persistence, patient satisfaction, and physician confidence—that often determine whether therapeutic innovations achieve meaningful impact outside research settings.

Another important contribution is the Delphi-based Spanish consensus developed by the ConverGHe Working Group (Castro-Feijóo et al.). This is the first Delphi consensus to provide national-level guidance on integrating LAGH into pediatric GHD care, offering practical recommendations based on current evidence and national expert opinion. It represents a useful document for physicians and a starting point for addressing questions regarding long-term efficacy and safety, as well as practical issues such as indications for treatment, timing of the switch from daily GH, dosing, and IGF-1 monitoring. The general concept is that LAGH is not superior to daily GH in terms of efficacy or safety but may offer advantages in adherence because weekly injections substantially reduce treatment burden.

The strongest rationale for LAGH may therefore be in children for whom daily injections are particularly challenging, especially adolescents and patients with needle anxiety, neurodiversity, multiple concomitant treatments, or adherence problems.

To date, LAGH has been approved for GHD, but several trials are exploring its use in other conditions, including children born small for gestational age (SGA) and syndromes such as Turner and Noonan syndromes. Zhang et al. described a late-pubertal boy with atypical Noonan syndrome treated with polyethylene glycol-conjugated rhGH (PEG-rhGH) combined with letrozole. In this context, combining GH stimulation with aromatase inhibition represents an innovative attempt to maximize residual growth potential by simultaneously enhancing growth velocity and delaying epiphyseal fusion. Although based on a single patient, this report highlights the potential value of a personalized approach to treatment.

LAGH may improve adherence through fewer injections, greater convenience, and reduced disruption of daily life. These benefits are intuitively appealing and are supported by patient-preference studies. However, the relationship between injection frequency, adherence, motivation, and ultimately clinical outcomes is more complex than initially assumed.

Gaudino et al. explored treatment burden and the still-unproven neuroendocrine mechanisms potentially related to motivation and adherence. Adherence is influenced by multiple interacting factors, including family support, health literacy, disease perception, socioeconomic conditions, healthcare accessibility, and patient engagement. Simply reducing injection frequency may remove one important barrier without necessarily addressing the broader determinants of long-term treatment persistence. Furthermore, hypotheses suggesting that long-acting formulations may exert direct neuroendocrine effects influencing motivation remain biologically intriguing but currently lack convincing clinical evidence. Distinguishing behavioral improvements resulting from simplified treatment regimens from intrinsic pharmacological effects will require carefully designed mechanistic studies.

Collectively, these articles underscore the increasing importance of generating high-quality real-world evidence alongside randomized clinical trials to evaluate long-term safety and efficacy, as well as quality of life, adherence, and outcomes extending into the transition to adult care, including final height and metabolic outcomes.

Nevertheless, enthusiasm for innovation should be accompanied by scientific rigor. Novel combination therapies, such as GH with aromatase inhibitors, require larger prospective studies before they can be recommended for widespread use.

These papers demonstrate how careful clinical observation, real-world evidence, expert consensus, and translational innovation can together contribute to improving the care of children with growth disorders. Despite evolving therapeutic approaches, the central objective remains unchanged: to enable every child with GHD to achieve not only optimal growth and height outcomes but also the best possible quality of life for the child and their family.

## References

[1] Schilbach K Clayton P Agrawal N Arlien-Søborg MC Backeljauw PF Biller BMK . Long-acting growth hormone: an updated Growth Hormone Research Society consensus statement. Eur J Endocrinol. (2026) 195:R73–96. doi: 10.1093/ejendo/lvag167 42676236

